# Organic and Inorganic Phosphorus Inputs Shape Wheat Productivity and Soil Bioavailability: A Microbial and Enzymatic Perspective from Long-Term Field Trials

**DOI:** 10.3390/microorganisms13112434

**Published:** 2025-10-23

**Authors:** Zhiyi Zhang, Yafen Gan, Fulin Zhang, Xihao Fu, Linhuan Xiong, Ying Xia, Dandan Zhu, Xianpeng Fan

**Affiliations:** 1 Institute of Plant Protection and Soil Fertilizer, Hubei Academy of Agricultural Sciences, Wuhan 430064, China; 2 Qianjiang Field Scientific Observation and Research Station for Agro-Environment, Ministry of Agriculture and Rural Affairs, Qianjiang 433100, China; 3 Hubei Engineering Research Center for Agricultural Non-point Source Pollution Control, Wuhan 430070, China; 4 Key Laboratory of Arable Land Conservation (Middle and Lower Reaches of Yangtze River), Ministry of Agri-culture and Rural Affairs, Huazhong Agricultural University, Wuhan 430070, China

**Keywords:** phosphorus fractions, enzyme activities, microbial communities, co-occurrence networks, wheat yield

## Abstract

Bioavailable phosphorus is essential for sustaining high crop productivity, yet excessive inorganic P fertilization often leads to P accumulation in stable soil forms, reducing utilization efficiency. Straw serves as an organic P source and enhances P availability by stimulating microbial activity. However, systematic studies on how organic P inputs (straw returning) and inorganic P fertilizers regulate soil bioavailable P through microbial and enzymatic processes remain limited. A 16-year field experiment was carried out in a rice–wheat rotation system, including five fertilization treatments: no fertilization (CK), optimized fertilization (OPT), increased N (OPTN), increased P (OPTP), and optimized fertilization combined with straw mulching/returning (OPTM). This study evaluates the impacts of long-term organic and inorganic P sources on soil P fractions, extracellular enzyme activities, and the composition of microbial communities, alongside their collective contributions to crop yield. In this study, based on soil samples collected in 2023, we found that fertilization led to significant increases in Citrate-P and HCl-P, enhanced the activities of β-1,4-glucosidase (BG), β-D-cellobiosidase (CBH), and β-1,4-N-acetylglucosaminidase (NAG), and altered both microbial diversity and co-occurrence network complexity. The OPTM treatment showed the highest yield and improved microbial diversity and network complexity, with Enzyme-P, Citrate-P, and HCl-P increasing by 62.64%, 11.24%, and 9.49%, and BG, CBH, and NAG activities rising by 22.74%, 40.90%, and 18.09% compared to OPT. Mantel tests and random forest analyses revealed significant associations between microbial community and biochemical properties, while partial least squares path modeling (PLS-PM) indicated that inorganic P source enhanced yield primarily through altering soil P dynamics and enzymatic processes, while microbial communities under organic P source acted as key mediators to increase crop productivity. These findings deepen insights into how microbial communities and enzymatic stoichiometry synergistically regulate phosphorus bioavailability and wheat yield, providing a theoretical basis for sustainable fertilization practices in rice–wheat rotation systems.

## 1. Introduction

In conventional agricultural systems, the widespread use of chemical fertilizers has long been considered essential for achieving high crop productivity. However, excessive fertilization has resulted in a series of environmental issues, including water eutrophication, elevated greenhouse gas emissions, and soil degradation [1,2], which increasingly threaten the sustainability of crop production systems. Therefore, optimizing fertilization strategies while maintaining crop productivity is crucial for promoting the sustainability of agriculture systems [3]. Fertilization generally leads to phosphorus accumulation in soils, but a large proportion of this phosphorus becomes stabilized through fixation by soil minerals, thereby limiting its availability to crops [4]. In contrast, as an organic phosphorus source, straw contributes by enhancing phosphorus mobilization and soil nutrient cycling [5,6]. Therefore, elucidating the variations in phosphorus fractions and crop yield under different P sources is of great importance for sustaining crop production.

Resource limitations are the primary regulators of enzyme activity and soil microbial metabolism [7]. N and P serve as key limiting macronutrients and play fundamental roles in cellular biosynthesis, energy metabolism, and other microbial functioning [8,9,10]. However, the utilization efficiency of N and P by crops remains low, particularly for phosphorus, with typical use efficiencies below 20% [11,12]. The remainder is either lost through various pathways or transformed into less bioavailable forms that accumulate in the soil [13]. In agroecosystems, N and P availability directly affects soil microbial functions and nutrient cycling, both of which are vital for plant growth and the maintenance of crop yields [14]. Organic and inorganic fertilizers exert different effects on soil enzyme activities and microbial communities. Organic inputs often stimulate enzyme activities by providing substrates and improving soil structure, whereas long-term chemical fertilization application may eventually reduce enzyme activity and even disrupt microbial communities [15,16].

Recent studies have demonstrated that fertilization exerts a substantial influence on soil phosphorus dynamics, enzyme activity, and microbial functions, ultimately enhancing crop yield [4,17]. Enzyme activities and community composition play pivotal roles in nutrient cycling, thereby having a substantial impact on soil quality and ecological functioning [18,19,20]. While shifts in microbial community composition and enzyme activity are known to mediate nutrient cycling, the mechanistic pathways by which organic and inorganic phosphorus sources regulate bioavailable P via microbial and enzymatic processes remain poorly understood [21,22]. Incorporation of crop straw has been widely reported to stimulate enzyme activities and enhance bacterial and fungal richness and diversity, leading to improved soil fertility [23,24]. Enzymes mediate key microbial metabolic activities, influencing microbial community composition and the formation of co-occurrence networks. Meanwhile, networks play pivotal roles in maintaining soil stability, nutrient cycling, and ecosystem resilience [25,26]. Understanding the structure and interactions within these networks is therefore crucial for linking microbial community composition to soil ecosystem functioning. In recent years, microbial co-occurrence network analysis has emerged as an effective approach to investigate interactions among soil microbes and their responses to environmental shifts [27]. Accumulating evidence shows that network structures are shaped by fertilization practices and tillage regimes [28,29].

High crop yields must be achieved without compromising environmental sustainability, which requires the optimized management of organic and inorganic phosphorus inputs; however, the effects of different fertilization regimes on yield and soil phosphorus fractions, as well as the underlying enzymatic and microbial mechanisms, remain poorly understood. To address this gap, we conducted a long-term field experiment in Qianjiang City, Hubei Province. Our objectives were to assess how different fertilization regimes influence soil P fractions, extracellular enzyme activities, and microbial community composition, and to elucidate the enzymatic and microbial mechanisms that drive phosphorus turnover and yield improvement. Achieving high crop yields while maintaining environmental sustainability requires optimized management of both organic and inorganic phosphorus sources. Although long-term fertilization promotes crop productivity, its mechanistic effects on soil phosphorus bioavailability, enzymatic activity, and microbial communities remain incompletely understood. In this long-term field experiment conducted in Qianjiang City, Hubei Province, we examined the responses of soil P fractions, extracellular enzymes, and microbial communities to various fertilization regimes, and investigated the enzymatic and microbial mechanisms underlying phosphorus turnover and crop yield enhancement under different P sources. The study tested the following hypotheses: (1) fertilization alters crop yield, soil P fractions, enzyme activity, and microbial community, and (2) organic and inorganic P sources differentially regulate yield, with inorganic P primarily affecting soil P dynamics and enzymatic processes and organic P promoting yield through microbially mediated pathways.

## 2. Materials and Methods

### 2.1. Study Site and Experimental Design

The long-term field experiment was initiated in 2007 at the National Station for Qianjiang Agro-Environment in Haokou Town, Qianjiang City, Hubei Province, China (30°22′55.1″ N, 112°37′15.4″ E). The sampling belonged to an alluvial plain, with a subtropical monsoon climate, an average annual temperature of 16.1 °C, and mean precipitation of 1100–1300 mm. Soil was classified as fluvo-aquic, developed from river alluvial deposits, with a deep soil layer and light texture. The cropping system is rice–wheat rotation. The initial physicochemical properties of the 0–20 cm soil layer were as follows: pH = 7.10; soil organic matter, 20.62 g kg^−1^; total N, 1.53 g kg^−1^; total P, 0.88 g kg^−1^; available P, 19.16 mg kg^−1^; and available K, 59.10 mg kg^−1^.

The study was arranged in a randomized complete block format with four replicates (6.0 m × 3.2 m plots) and comprised five treatments: (1) the control (CK)—no fertilizer applied; (2) OPT, optimized fertilizer—the amount of fertilizer application depended on the research results in recent years; (3) OPTN, increased nitrogen fertilizer—increased nitrogen application by 50% based on optimized fertilization treatments; (4) OPTP, increased phosphate fertilizer—increased phosphate application by 50% based on optimized fertilization treatments; (5) OPTM, optimized fertilizer + straw mulching/returning—optimized fertilization combined with straw mulching/returning to the field. All fertilization treatments are based on the optimized inorganic fertilization rate of OPT, and the other treatments include additional nutrient inputs (N, P, or straw) in addition to this baseline. Similar treatments were carried out during both cropping seasons, with N, P, and K fertilizer and straw application rates provided in Table S1.

The fertilizers for N, P, and K consisted of urea, calcium superphosphate, and potassium chloride, respectively. Each season, 60% of N and 100% of P and K were incorporated into the soil as basal fertilizer prior to sowing, while the remaining 40% of N was supplied as topdressing. For the consistency and rigor in the plot experiments, only the root stubble was retained after harvest, and all the returned straw originated from the same area as the designated straw return plots, under the same crop rotation and management practices. The experimental plots were 19.2 m^2^ in size, separated by ridges between the plots, with fixed drainage and irrigation ditches (ditch width 40 cm^2^) between the groups, and each plot could be drained independently.

### 2.2. Soil Sampling and Analysis

After 16 years of continuous field management, soil samples were collected in early April 2023. A composite sample was obtained for each plot by mixing three randomly collected soil cores (0–20 cm depth). All samples were preserved on dry ice during transport to the laboratory. Stones and plant residues were manually removed with forceps, followed by sieving through a 2 mm mesh. The homogenized material was then partitioned into three subsamples: one air-dried for physicochemical properties determination, another stored at 4 °C for extracellular enzyme assays, and the remainder frozen at −20 °C for microbial community analysis.

The soil pH was measured by a pH meter with a water-to-soil ratio of 2.5:1. Soil organic matter (SOM) was quantified using the K_2_Cr_2_O_7_-FeSO_4_ oxidation method. Total nitrogen (TN) was estimated using the Kjeldahl method, while total phosphorus (TP) was determined using the molybdenum blue method. The available phosphorus (AP) was extracted using NaHCO_3_ and measured colorimetrically, while available potassium (AK) was obtained using CH_3_COONH_4_ extraction and quantified by flame photometry. Water-extractable organic carbon (WEOC) and NO_3_^−^–N were assessed using a total organic carbon analyzer and a continuous-flow autoanalyzer, respectively. Soil microbial biomass carbon (MBC) and nitrogen (MBN) were assessed through chloroform fumigation extraction with K_2_SO_4_, while soil microbial biomass phosphorus (MBP) was quantified same fumigation approach coupled with NaHCO_3_ extraction [30,31].

### 2.3. Measurement of Soil Bioavailable Phosphorus Components

The bioavailable-based P (BBP) method identifies four distinct soil P fractions by employing extraction with CaCl_2_ (0.01 M), citrate (0.01 M), phosphatase (0.2 units mL^−1^), and HCl (1.0 M) [32,33]. In total, 0.5 g of soil was mixed with 10 mL of designated extractant in separate centrifuge tubes and agitated at 180 rpm for 3 h. After centrifugation (4000× *g*, 25 °C, 30 min), the supernatants were collected. All samples were performed via the malachite-green colorimetric method at 630 nm with a multifunctional microplate reader (PerkinElmer, Waltham, MA, USA). Since commercial phytase contains phosphorus, it was dialyzed at 4 °C for five days to remove background phosphorus.

### 2.4. Analysis of Soil Extracellular Enzyme Activities

Extracellular enzyme activities (EEAs) involved in C, N, and P cycling were determined using a fluorescence microplate assay [34,35]. The activities of β-1,4-glucosidase (BG), β-D-cellobiosidase (CBH), β-1,4-N-acetylglucosaminidase (NAG), L–leucine aminopeptidase (LAP), and alkaline phosphatase (ALP) were quantified using fluorogenic substrates labeled with 4-methylumbelliferone (MUB) or 7-amino-4-methylcoumarin (AMC). Fresh soil (0.5 g) was homogenized in acetate buffer (50 mL, 50 mM), and aliquots (200 µL) were placed into 96–well plates for assay, blank, and quench standards. Substrate solutions (50 µL, 200 µM) were added to assay wells, acetate buffer (50 µL) was added to blank wells, and MUB or AMC standards (50 µL, 10 µM) were used for quenching correction. Negative control and reference standard wells each contained acetate buffer (200 µL), supplemented with 50 µL substrate or standard solution, respectively. After a 3 h incubation in the dark at 25 °C for 3 h, fluorescence was recorded (excitation: 365 nm; emission: 450 nm). Enzyme activities (nmol h^−1^ g^−1^) were calculated after negative control and quench corrections.

### 2.5. Vector Analysis of Soil Extracellular Enzyme Activities

Vector analysis was employed to assess C, N, and P limitations in soil microbial metabolism based on extracellular enzyme stoichiometry [36]. Vector length reflects relative balance between C and nutrient acquisition, with longer length signifying stronger C limitation, whereas the vector angle indicates the relationship between P and N acquisition, where values > 45° suggest P limitation and <45° imply N limitation. The following formula was used for calculation:

(1) $$\mathrm{Vector}\;\mathrm{length}=\sqrt{x^{2}+y^{2}}$$

(2) $$\mathrm{Vector}\;\mathrm{angle}\;({}^{\circ})=\mathrm{DEGREES}\left(\mathrm{ATAN}2\left(x,y\right)\right)$$

x represents the ratio of C- to P-acquiring enzymes, calculated as (BG + CBH) divided by the sum of (BG + CBH) and AP; y denotes the proportion of C- to N-acquiring enzymes, defined as (BG + CBH) over the sum of (BG + CBH) and (NAG + LAP).

E_C:N_, E_C:P_, and E_N:P_ were calculated as follows [37]:

(3) $$E_{C:N}=\ln\left(\mathrm{BG}+\mathrm{CBH}\right)/\ln\left(\mathrm{NAG}+\mathrm{LAP}\right)$$

(4) $$E_{C:P}=\ln\left(\mathrm{BG}+\mathrm{CBH}\right)/\ln\left(\mathrm{ALP}\right)$$

(5) $$E_{N:P}=\ln\left(\mathrm{NAG}+\mathrm{LAP}\right)/\ln\left(\mathrm{ALP}\right)$$

### 2.6. Soil DNA Extraction, High-Throughput Sequencing, and Co-Occurrence Analyses

Soil DNA was isolated from 0.5 g of fresh soil using the Fast DNA SPIN soil DNA isolation kit (MP Biomedicals, Santa Ana, CA, USA). The concentration and purity of the isolated DNA were then evaluated with a NanoDrop 2000 UV–Vis spectrophotometer (Thermo Scientific, Wilmington, DE, USA). Extracted DNA samples were stored at −80 °C until subsequent analyses. Quantitative PCR (qPCR) targeting bacterial and archaeal 16S rRNA genes as well as fungal 18S rRNA genes was conducted using SYBR Premix Ex Taq (Takara Bio, Dalian, China) on a LightCycler 480 instrument (Roche Diagnostics, Mannheim, Germany) to determine absolute gene abundances. For microbial community profiling, PCR amplification employed previously validated primers detailed in earlier studies [38,39,40]. The purified amplicons were subjected to paired-end sequencing (2 × 300 bp) on the Illumina MiSeq platform (Illumina, San Diego, CA, USA). All sequencing services and technical assistance were provided by Shanghai Majorbio Bio-pharm Technology Co., Ltd. (Shanghai, China).

A co-occurrence network integrating bacterial, fungal, and archaeal communities was established based on Spearman’s correlation coefficients (r > 0.75, *p* < 0.01) calculated in R 4.3.1. The network was visualized and topological features were analyzed with Gephi v0.10.1. The average of edges per node was used to measure network complexity [41]. Nodes were classified into four distinct topological roles according to within-module connectivity (Zi) and among-module connectivity (Pi) values [42]: peripherals (Zi < 2.5, Pi < 0.62), connectors (Pi > 0.62), module hubs (Zi > 2.5), and network hubs (Zi > 2.5, Pi > 0.62).

### 2.7. Statistical Analysis

Alpha diversity indices were computed using QIIME v.1.8.0 to assess microbial diversity within each sample. One-way analysis of variance (ANOVA) was performed to assess soil and microbial variables across treatments. Duncan’s test was used to detect significant differences (*p* < 0.05). The impacts of SOM and AP on crop yield were evaluated using a general linear model (GLM). Pearson correlation analysis was applied to examine relationships among soil properties and microbial communities. Beta diversities were visualized and examined through non-metric multidimensional scaling (NMDS) based on weighted UniFrac distances with the “vegan” package (version 2.6-4) of R 4.3.1. Mantel tests were conducted to evaluate correlations between microbial composition and soil variables. To identify the key predictors of crop yield, a random forest model was developed using the “randomForest (4.7-1.1)” and “rfPermute (v2.5.2)” packages, with permutation importance used to assess variable contributions. Partial least squares path modeling (PLS-PM) was employed to investigate factors influencing yield, providing a comprehensive view of the mechanistic relationships.

## 3. Results

### 3.1. Soil Chemical Properties and Microbial Biomass

Fertilization led to significantly lower soil pH and higher contents of SOM, TN, TP, AP, and MBN compared to CK treatments (*p* < 0.05) (Table 1). The effects of organic and inorganic P sources on soil properties differed among treatments. OPTM exhibited the highest SOM (33.16 g kg^−1^), TN (1.63 g kg^−1^), AK (111.21 mg kg^−1^), AN (123.03 mg kg^−1^), WEOC (180.74 mg kg^−1^), and MBN (43.19 mg kg^−1^), whereas OPTP was characterized by higher TP, AP, and NO_3_^−^–N contents. Moreover, AP was significantly higher in OPTP compared with other fertilization treatments (*p* < 0.05).

### 3.2. Variation in Crop Yield

**Figure 1:**
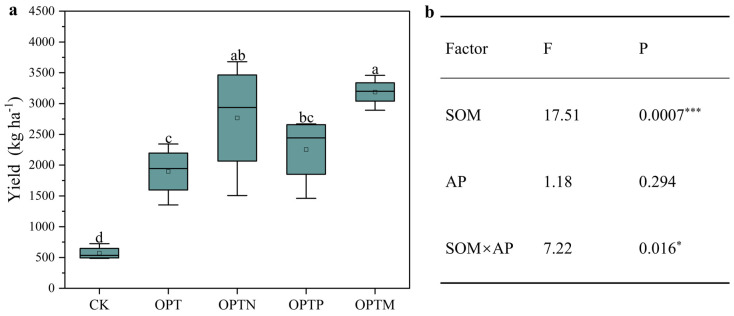
Impact of fertilization on crop yield and summaries of general linear model (GLM). (**a**) Yield; (**b**) interaction effect of SOM and AP on yield based on GLM. Vertical bars indicate the standard errors. Different letters indicate significant differences between treatments (Duncan, *p* < 0.05). *, *p* < 0.05; ***, *p* < 0.001. Boxplots represent the distribution of the data, and the point within each box denotes the mean.

Fertilizer application significantly enhanced crop yield compared to CK treatment (Figure 1a), with the OPTM treatment producing the highest yield. Compared to the OPT treatment, the yield under the OPTN, OPTP, and OPTM treatments increased by 45.78%, 18.80%, and 68.04%, respectively. The general linear model (Figure 1b) revealed that SOM had a highly significant positive effect on yield (*p* < 0.001), while AP was not statistically significant (*p* = 0.294). However, the interaction between SOM and AP showed a significant effect on yield (*p* = 0.016), suggesting a potential synergistic effect between the two factors in enhancing crop production.

### 3.3. Soil P Fractions

**Figure 2:**
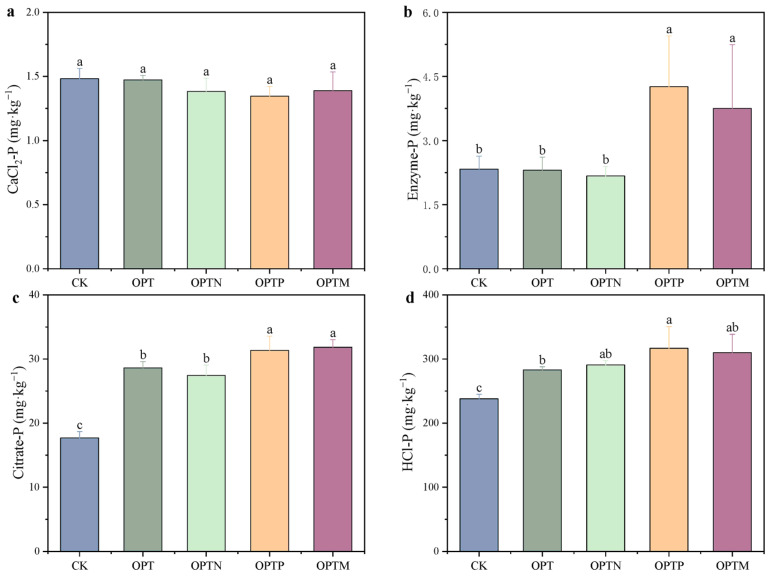
Effects of various treatments on bioavailable phosphorus components. (**a**) CaCl_2_-P; (**b**) Enzyme-P; (**c**) Citrate-P; (**d**) HCl-P. Vertical bars represent standard errors. Different letters indicate significant differences between treatments (Duncan, *p* < 0.05).

Fertilization exhibited varying effects on different forms of bioavailable phosphorus, but CaCl_2_-P showed no significant variation among treatments (Figure 2). In contrast, the contents of Enzyme-P, Citrate-P, and HCl-P varied substantially across treatments, ranging from 2.17 to 4.26 mg kg^−1^, 17.70 to 31.83 mg kg^−1^, and 238.02 to 316.84 mg kg^−1^, respectively. Compared with CK, fertilization increased Citrate-P by 54.97–79.82% and HCl-P by 18.90–30.18%, with significant enhancements observed in OPTP and OPTM treatments. Additionally, Enzyme-P in OPTP and OPTM increased by 82.84% and 60.63%, respectively, compared with CK. OPTM treatment increased Enzyme-P, Citrate-P, and HCl-P by 62.64%, 11.24%, and 9.49% compared to OPT. Both OPTP and OPTM treatments exhibited high concentrations across the four P fractions, highlighting their roles in promoting Enzyme-P and inorganic P (Citrate-P and HCl-P) availability.

### 3.4. Soil Extracellular Enzyme Activities, Stoichiometry, and Vector Characteristics 

**Figure 3:**
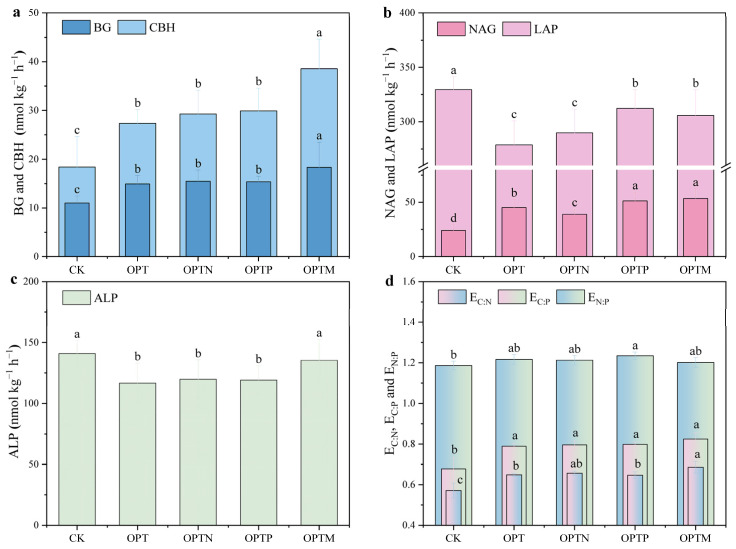
Soil extracellular enzyme activities and stoichiometric characteristics. (**a**) BG and CBH represent β-1,4-glucosidase and β-D-cellobiosidase; (**b**) NAG and LAP represent β-1,4-*N*-acetylglucosaminidase and L-leucine aminopeptidase; (**c**): ALP represents alkaline phosphatase; (**d**): E_C:N_, E_C:P_, and E_N:P_. Significant differences (*p* < 0.05) between treatments are indicated with different lowercase letters.

Fertilization significantly influenced both activities and stoichiometric traits of soil extracellular enzymes (Figure 3). C-acquiring enzyme (BG and CBH) exhibited consistent trends across treatments (Figure 3a), with BG exhibiting higher activity than CBH, especially in OPTM treatment. NAG activity was significantly enhanced in OPTP and OPTM compared to CK, while LAP and ALP activity declined with all fertilization treatments (Figure 3b,c). BG, CBH, and NAG activities in OPTM increased by 22.74%, 40.90%, and 18.09%, respectively, compared to OPT. E_C:N_, E_C:P_, and E_N:P_ were lowest in CK. Both E_C:N_ and E_C:P_ reached their highest values under OPTM, while E_N:P_ was highest under OPTP.

**Figure 4:**
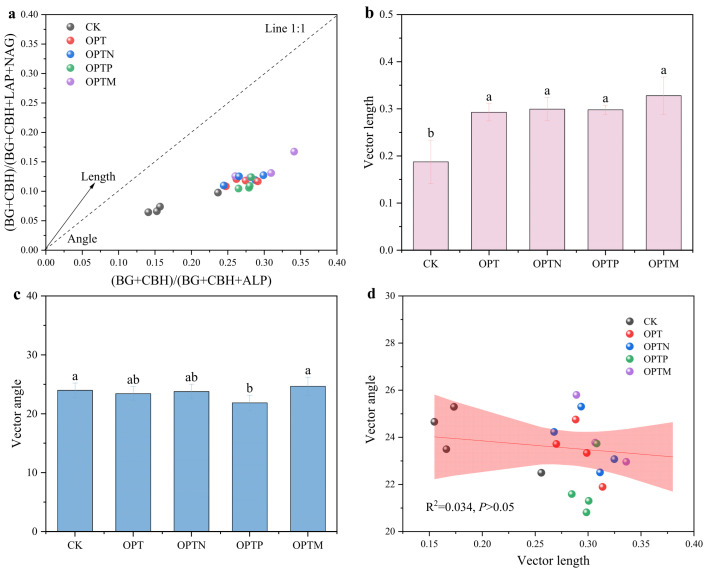
Ecoenzymatic vector model quantifying microbial resource limitation. (**a**) Ratio of C to N acquisition versus C to P acquisition; (**b**) vector length represents microbial C limitation; (**c**) vector angle represents microbial N or P nutrient limitation; (**d**) linear relationships between vector length and vector angle. Different letters indicate significant differences between treatments (*p* < 0.05, Duncan’s multiple range test). Linear regression was used to identify the relationship between microbial C and N limitation.

Based on the stoichiometric traits of extracellular enzyme activities, the observed data points fell beneath the 1:1 line (Figure 4a), suggesting that the microbial community was N-limited. Fertilization significantly increased the vector length (Figure 4b), indicating intensified microbial C limitation, with the longest vector length under the OPTM. The vector angles across treatments ranged from 21.86° to 24.67° (Figure 4c), with OPTP showing a significantly lower angle than CK, indicating the mitigation of microbial nitrogen limitation. Additionally, linear regression analysis revealed a negative relationship between vector angle and length, suggesting a decrease in microbial N limitation accompanied by increased C limitation (Figure 4d), although this correlation was not statistically significant.

### 3.5. Soil Microbial Community and Associations with Soil Properties

**Figure 5:**
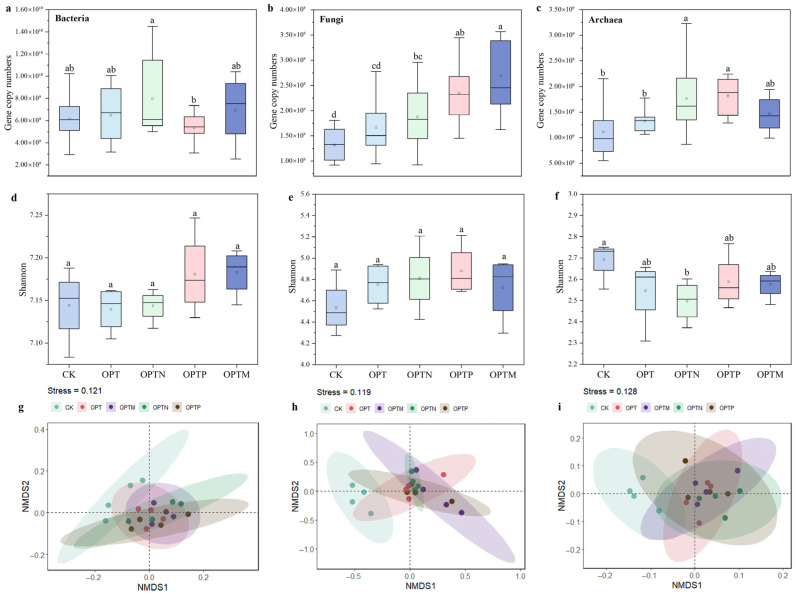
Soil microbial community diversity under different fertilization treatments. (**a**–**c**) Box plots of gene copy numbers for bacteria (**a**), fungi (**b**), and archaea (**c**); (**d**–**f**) α-diversity (Shannon index) of bacteria (**d**), fungi (**e**), and archaea (**f**); (**g**–**i**) nonmetric multidimensional scaling analysis (NMDS) based on Bray–Curtis distances showing β–diversity of bacteria (**g**), fungi (**h**), and archaea (**i**). Different letters indicate significant differences among different fertilization treatments (Duncan, *p* < 0.05). Boxplots represent the distribution of the data, and the point within each box denotes the mean.

Fertilization significantly affected microbial gene copy numbers, with distinct responses observed among bacteria, fungi, and archaea (Figure 5a–c). Compared with CK, the OPTN, OPTP, and OPTM significantly increased fungal and archaeal gene copy numbers. The order of microbial alpha diversity was bacteria > fungi > archaea (Table S2). Fertilization increased the bacterial and fungal Shannon index, especially in OPTP and OPTM (Figure 5d–f), whereas the Shannon index of archaea decreased following fertilization. NMDS results further indicated significant differences between CK and fertilization treatments, demonstrating that fertilization substantially altered microbial community structure (Figure 5g–i).

**Figure 6:**
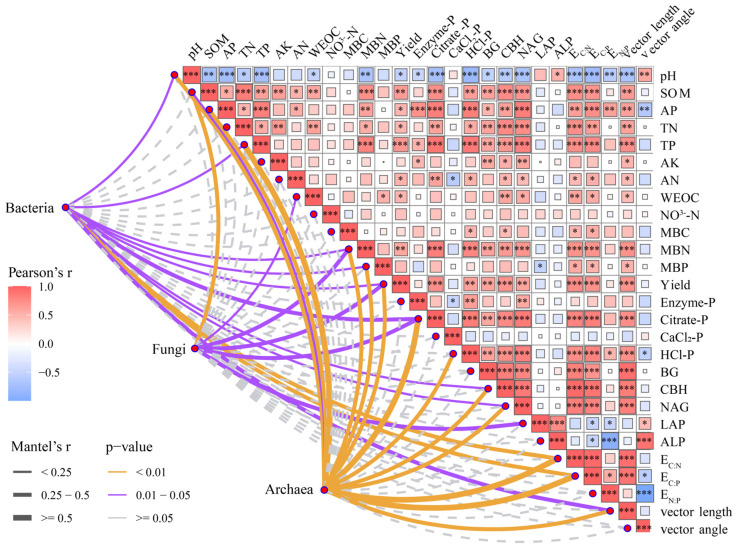
Relationships between microbial community composition and environmental factors based on Mantel tests. *, *p* < 0.05; **, *p* < 0.01; ***, *p* < 0.001.

The bacterial community was primarily composed of Proteobacteria, Acidobacteriota, and Chloroflexi, while Ascomycota prevailed in the fungal community, followed by Basidiomycota, Mortierellomycota, and Rozellomycota (Figure S1). Crenarchaeota was the predominant archaeal phylum and increased with fertilization, whereas Euryarchaeota and Halobacterota declined (Figure S1c). Mantel tests revealed significant correlations between microbial community structures (especially bacterial and archaeal communities) and various soil parameters, including MBN, MBP, yield, Citrate-P, HCl-P, CBH, NAG, LAP, E_C:N_, E_C:P_, and vector length (Figure 6). Notably, yield was significantly positively correlated with TP, AP, MBN, Citrate-P, HCl-P, BG, CBH, NAG, E_C:N_, E_C:P_, and vector length. Correlation analysis further demonstrated that fungal gene copy number, Proteobacteria, Mortierellomycota, Rozellomycota, and Crenarchaeota were strongly positively correlated with soil properties and crop yield (Figure S2). 

### 3.6. Microbial Co-Occurrence Networks

**Figure 7:**
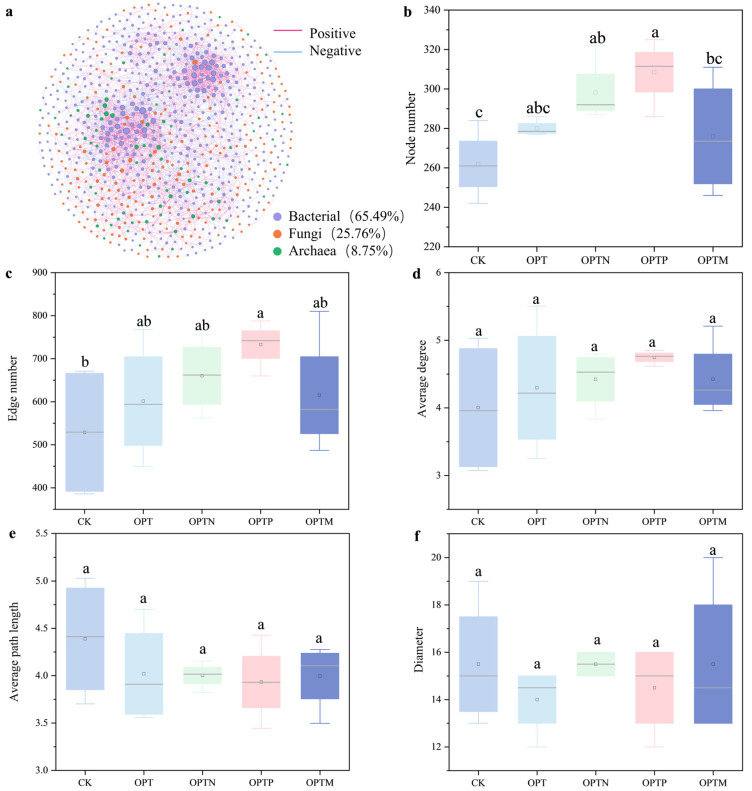
Co-occurrence network of bacteria, fungi, and archaea. Multiple indices were used to estimate the complexity of co-occurrence networks. (**a**) Co-occurrence network of bacteria, fungi, and archaea; (**b**–**f**) complexity indices of subnetworks. Different letters indicate significant differences among different fertilization treatments (Duncan, *p* < 0.05). Boxplots represent the distribution of the data, and the point within each box denotes the mean.

The network analysis resulted in a network with 823 nodes and 2698 edges, with a modularity of 0.596 and an average clustering coefficient of 0.294. Bacterial, fungal, and archaea taxa contributed 65.49%, 25.76%, and 8.75% of the nodes, respectively (Figure 7). By constructing subnetworks, the impact of fertilization on the complexity of soil subnetworks was assessed. Fertilization increased the number of nodes and edges (*p* < 0.05) and the average degree (*p* > 0.05) but reduced the average path length. These findings indicate that fertilization enhances soil subnetwork complexity primarily by increasing nodes and edges while shortening average path length and diameter.

**Figure 8:**
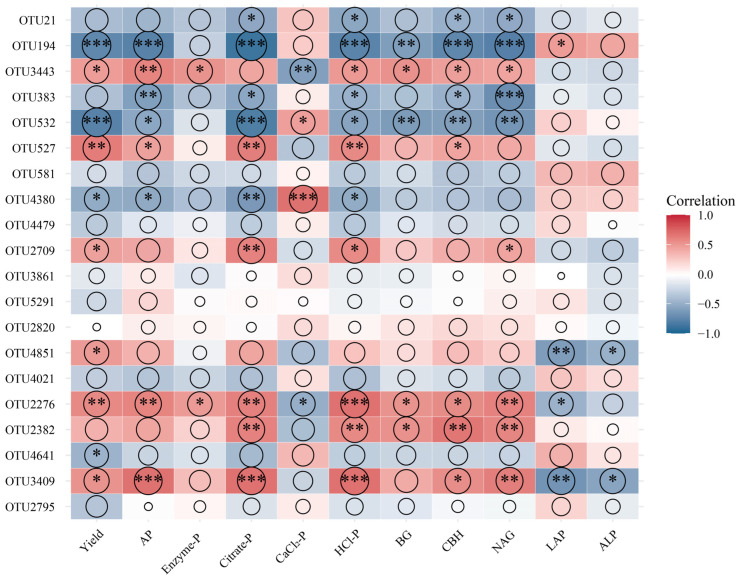
Correlations between keystone taxa (network hubs and module hubs), soil properties and yield. *, *p* < 0.05; **, *p* < 0.01; ***, *p* < 0.001. Circle size represents the strength of correlation.

Based on network topological metrics, a bacterial OTU affiliated with Actinobacteriota was identified as a network hub (Figure S3), and 19 nodes were recognized as module hubs, including 13 bacterial, 4 fungal, and 3 archaeal taxa. The putative keystone bacterial taxa primarily belonged to Proteobacteria, Nitrospirota, Acidobacteriota, Chloroflexi, and Bacteroidota, whereas fungal keystone taxa were Basidiomycota and Ascomycota. Archaeal keystone taxa were primarily affiliated with Crenarchaeota and Halobacterota. Correlation analysis showed that OTU3409, OTU2276, OTU4851, OTU2709, OTU527, and OTU3443 were significantly correlated with both soil properties and yield (Figure 8). For instance, OTU2276 and OTU2382 were positively correlated with Citrate-P, HCl-P, BG, CBH, and NAG, while OTU532 and OTU194 were negatively associated with yield and most soil properties.

### 3.7. Effects of Soil Properties and Microbial Community Structure on Crop Yield

**Figure 9:**
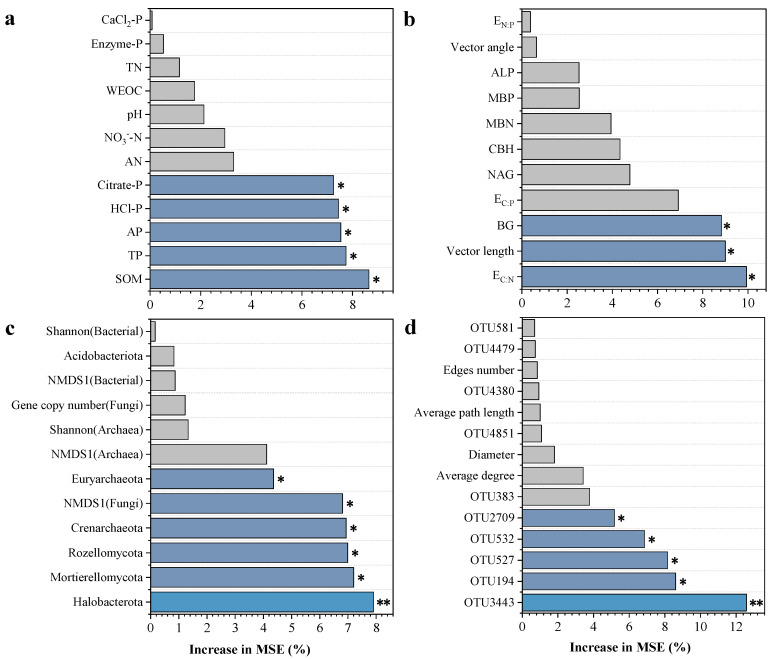
Predictions of factors contributing to crop yield based on random forest regression analysis. (**a**) Soil physicochemical properties and P fractions; (**b**) soil microbial biomass and enzyme stoichiometry; (**c**). soil microbial community structure; (**d**) microbial co-occurrence networks. Percentage increases in the mean square error (MSE%) values imply the importance of these predictors. Asterisks represent significance predictors at *p* < 0.05 (*) and *p* < 0.01 (**).

**Figure 10:**
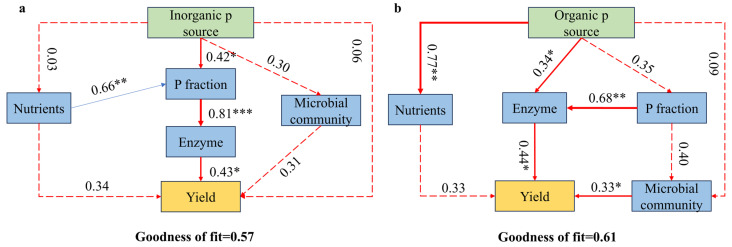
Path analysis diagrams based on the partial least squares path model (PLS-PM). PLS-PM describing the direct and indirect effects of inorganic P sources (**a**) and organic (**b**) P sources on crop yield. The red and blue arrows indicate significant positive and negative effects (*p* < 0.05), respectively, whereas dashed arrows indicate no significant relationships. The significance levels of each predictor are *p* < 0.05 (*), *p* < 0.01 (**), and *p* < 0.001 (***).

A random forest regression analysis was employed to identify and rank the key factors influencing crop yield (Figure 9). Among soil physicochemical properties and P fractions, SOM, TP, AP, HCl-P, and Citrate-P were identified as significant predictors of crop yield (*p* < 0.05). Regarding enzymatic stoichiometric traits, the E_C:N_, vector length, and BG emerged as the most influential variables (*p* < 0.05). Within the microbial community, Halobacterota was the most influential taxon in predicting yield, followed by Mortierellomycota, Rozellomycota, Crenarchaeota, NMDS1 (Fungi), and Euryarchaeota (*p* < 0.05). Within the microbial co-occurrence network analysis, OTU3443 was the most critical predictor of yield, followed by OTU194, OTU527, OTU532, and OTU2709. The PLS-PM model was applied to evaluate the effects of long-term fertilization, P fractions, soil enzymatic activity, and microbial communities on crop yield (Figure 10). This model indicates that inorganic P source primarily enhances crop yield indirectly by altering phosphorus fractions and modulating enzyme activities. In contrast, an organic source of P (straw returning) highlights the pivotal role of microbial community diversity and significantly improves soil nutrient status. 

## 4. Discussion

### 4.1. Effects of Organic and Inorganic P Sources on Soil P Fractions and Wheat Yield

In this study, fertilization significantly enhanced wheat yield. Notably, OPTN and OPTM treatments produced the highest yield, highlighting the potential of combined nutrient management for sustaining high crop productivity (Figure 1). In contrast, despite exhibiting the highest levels of TP and AP, OPTP resulted in a relatively limited increase in yield. This suggests that high levels of P application may have a limited impact on yield, likely because unabsorbed phosphorus becomes immobilized in the soil [43,44]. Previous studies have demonstrated that appropriate P application can promote root elongation and enhance phosphorus uptake, which are critical factors for achieving maximum crop yield [45,46]. The findings showed that fertilizer application significantly raised soil TP and AP (Table 1), particularly in OPTP and OPTM treatments. This effect is primarily attributed to increased phosphorus inputs and the addition of organic materials, with straw-derived carbon inputs further promoting phosphorus activation and plant uptake [47]. 

The BBP method effectively evaluates biological mechanisms, reflecting soil P availability and acquisition strategies [48]. In our study, fertilization, especially when combining organic and inorganic P sources, stimulated the turnover of bioavailable phosphorus (Figure 2), which primarily increased the contents of CaCl_2_-P, Citrate-P, and HCl-P. Meanwhile, Citrate-P, and HCl-P represent potentially available phosphorus forms, and their dynamic changes are of critical importance for the sustainable management of agricultural soil health [32,49]. Combined organic and inorganic P inputs improve soil physicochemical properties, stimulate microbial proliferation and root development, and enhance the secretion of compounds such as organic acids and phosphatases, thereby mobilizing various forms of P [33,50]. 

### 4.2. Microbial and Enzymatic Mediation of Phosphorus Turnover Under Fertilization

Soil extracellular enzymes play a pivotal role in nutrient cycling and metabolic processes within soil ecosystems [26,51]. In this study, fertilization significantly enhanced the activities of BG, CBH, and NAG, which were positively correlated with soil Citrate-P and HCl-P (Figure 3 and Figure 6). The possible mechanism for improved enzyme activity can be attributed to fertilization promoting microbial biomass, stimulating root exudation, and improving nutrient availability [52]. Vector analysis indicated that microbes were primarily limited by C and N, and the increased activity of C- and N-acquiring enzymes, rather than P-acquiring enzymes, represents a key strategy to sustain microbial growth and metabolism [36,53]. Enzyme-mediated organic matter decomposition facilitates nutrient release and phosphorus turnover, and straw incorporation provides additional substrates that further enhance this process [54,55]. The alleviation of microbial N limitation in OPTP treatment likely reflects the pivotal role of P in cellular metabolism, as sufficient P promotes microbial growth and metabolic activity by directly participating in essential life processes, thereby enhancing nitrogen use efficiency [6,56]. Straw returning simultaneously increased microbial C and N limitation (Figure 4b,c), likely due to its high C/N ratio and disturbance of soil nutrient balance. The high C input provides abundant substrate but limited N, forcing microbes to consume more soil N to sustain growth and metabolism, thereby enhancing N limitation. Meanwhile, the demand for energy and biomass synthesis drives increased decomposition of SOC, intensifying C limitation [57]. 

Long-term fertilization significantly changes soil physicochemical properties, thereby reshaping the structure of microbial communities [58,59]. The organic fertilizers have been shown to enhance bacterial diversity, thereby strengthening microbial community resistance to external disturbances [60]. Our study also found the highest bacterial Chao1 and Shannon indices in OPTM treatment (Figure 5). Proteobacteria and Acidobacteriota were the dominant bacterial phyla in the soil (Figure S1). Fertilization increased the abundance of copiotrophic Proteobacteria that rapidly respond to nutrient inputs, thereby accelerating soil C mineralization and resource turnover [61,62]. In fungal communities, fertilization decreased the abundance of Ascomycota but increased that of Mortierellomycota and Rozellomycota. Previous studies have suggested that Ascomycota are copiotrophic fungi with r-strategy traits, and thus their abundance could increase under nutrient enrichment [63]. The discrepancy with our findings may be attributed to intensified competition for resources that limited their enrichment [64]. Fertilization also increased the abundance of Crenarchaeota, which serve as a biomarker for pelagic ammonia-oxidizing archaea (AOA). Microbial keystone taxa are those that exert considerable effects on microbiome structure and function [60,65]. In this study, the keystone bacterial taxa belonged to six phyla (Table S3). The keystone fungal groups belonged to Ascomycota and Basidiomycota, while the archaeal keystone taxa were Crenarchaeota and Halobacterota. Previous studies have reported that such keystone taxa are critical to soil nutrient cycling, SOM mineralization and nitrogen transformations [27,66,67].

Correlation analysis revealed that soil bioavailable phosphorus was significantly positively associated with Proteobacteria, Mortierellomycota, Rozellomycota, and Crenarchaeota (Figure S2), suggesting that these microbial groups play key roles in phosphorus turnover. Copiotrophic Proteobacteria rapidly respond to nutrient inputs, accelerating carbon and nutrient cycling, while Mortierellomycota can synthesize and secrete low-molecular-weight organic acids, facilitating organic P mineralization. Crenarchaeota, as ammonia-oxidizing archaea, may indirectly facilitate P availability through their roles in nitrogen transformations. 

### 4.3. Integrated Effects of P Fractions, Enzyme Activities, and Microbial Communities on Wheat Yield

Long-term fertilization is broadly acknowledged as a key factor regulating crop yield through its profound impacts on soil properties and microbial communities [2,68]. In this study, we demonstrated that both organic and inorganic P inputs significantly influenced wheat yield by modulating soil P fractions, enzymatic activities, and microbial community composition. In addition to AP and TP, significant positive correlations were found between yield and both Citrate-P and HCl-P (Figure 6). These results suggest that potential bioavailable inorganic P pools provide sustained nutrient release throughout the crop growth cycle and serve as key determinants of productivity [49,69]. Random forest analysis further confirmed that AP, TP, Citrate-P, and HCl-P were among the most important predictors of wheat yield (Figure 9). Soil enzyme activities represent crucial mediators linking microbial dynamics and nutrient cycling [70,71]. In our study, BG, CBH, and NAG showed positive correlations with yield (Figure 6), reflecting their roles in decomposing SOM and facilitating nutrient mineralization [72]. The observed correlations between enzyme activities and soil nutrients highlight the importance of soil enzymes in maintaining soil productivity. 

Mantel tests revealed that bacterial, fungal, and archaeal communities were significantly associated with yield and various soil properties (Figure 6). Correlation analyses further showed that the phyla Proteobacteria, Rozellomycota, and Crenarchaeota, along with bacterial gene copy numbers and archaeal Shannon indices, were positively correlated with yield (Figure S2). Soil microorganisms contribute to nutrient acquisition and plant productivity by regulating nutrient availability [73]. Proteobacteria are widely recognized for their capabilities in P solubilization and the decomposition of SOM [61], while Crenarchaeota, as key ammonia-oxidizing archaea, play a pivotal role in nitrogen cycling [74]. These microbial taxa may enhance nutrient availability and improve rhizosphere conditions, thereby facilitating nutrient uptake and increasing yield.

The PLS-PM model further indicated that inorganic P source primarily enhanced yield indirectly by altering soil phosphorus dynamics and enzymatic processes, while organic P source increased yield through microbially mediated pathways. These findings highlight that organic P source (straw returning) enhances crop yield predominantly through the modulation of soil microbial communities and enzymatic nutrient transformation processes, rather than solely through nutrient supplementation. Notably, the OPTM treatment significantly increased gene copy numbers and Shannon diversity (Figure 5), and OTU3409, OTU2276, OTU4851, OTU2709, OTU527, and OTU3443 were positively correlated with yield (Figure 8). Previous studies have highlighted the critical role of keystone taxa in sustaining soil functional potential and determining wheat yield [2,75]. The input of organic P source and diverse microbial communities via straw returning likely contributes to soil functional stability, emphasizing the microbial pathways through which organic P source enhances yield [76].

### 4.4. Implications

This 16-year field investigation underscores the critical importance of integrating organic and inorganic phosphorus management to enhance soil fertility and crop productivity in rice–wheat rotation systems. While sole inorganic P application can increase yield, organic P inputs (straw returning) more effectively stimulate microbially mediated P transformations and key extracellular enzyme activities, constituting a pivotal microbial mechanism for yield improvement. These findings provide robust scientific guidance for agricultural practice: balanced application of organic and inorganic P can sustain high productivity while mitigating nutrient loss and environmental pollution. For farmers, adopting such fertilization strategies can improve nutrient-use efficiency and maintain long-term soil fertility. For policymakers, the results highlight the need for policy incentives and extension programs that promote balanced fertilization and recycling of organic resources. More broadly, this work offers solid evidence to support region-specific nutrient management strategies that reconcile food security with environmental sustainability.

## 5. Conclusions

Long-term (16-year) fertilization markedly influenced soil phosphorus fractions, enzyme activities, microbial community composition, and network complexity in a rice–wheat rotation system, collectively contributing to enhanced crop productivity. Inorganic P primarily enhanced yield indirectly by altering soil phosphorus dynamics and enzymatic processes, while organic P increased yield through microbially mediated pathways. Among the treatments, OPTM emerged as the most effective and environmentally sustainable strategy, as its yield benefits were mechanistically linked to elevated bioavailable P fractions (Enzyme-P, Citrate-P, and HCl-P), activation of C- and N-acquiring enzymes (BG, CBH, and NAG), and the enrichment of functional microbial taxa and keystone species. Key microbial taxa, including copiotrophic Proteobacteria, Mortierellomycota, Rozellomycota, and Crenarchaeota, played central roles in regulating phosphorus availability through both direct and indirect mechanisms, highlighting the critical role of microbial and enzymatic processes in sustaining soil fertility and crop productivity.

## Figures and Tables

**Table 1:** Soil chemical properties under different fertilization treatments.

Parameter	CK	OPT	OPTN	OPTP	OPTM
pH	8.01 ± 0.03 a	7.75 ± 0.04 b	7.67 ± 0.05 b	7.67 ± 0.11 b	7.70 ± 0.22 b
SOM (g kg^−1^)	22.23 ± 0.73 c	26.57 ± 1.93 b	27.45 ± 2.24 b	26.92 ± 1.34 b	33.16 ± 2.35 a
TN (g kg^−1^)	1.23 ± 0.10 c	1.42 ± 0.20 b	1.39 ± 0.03 bc	1.39 ± 0.10 bc	1.63 ± 0.04 a
TP (g kg^−1^)	0.67 ± 0.02 c	0.99 ± 0.05 b	1.03 ± 0.15 ab	1.15 ± 0.14 a	1.09 ± 0.04 ab
AK (mg kg^−1^)	82.64 ± 3.74 b	86.44 ± 4.82 b	79.72 ± 8.58 b	94.32 ± 23.91 ab	111.21 ± 12.84 a
AP (mg kg^−1^)	5.77 ± 5.84 d	48.69 ± 8.85 c	39.89 ± 6.07 c	92.63 ± 9.03 a	62.84 ± 9.53 b
AN (mg kg^−1^)	80.30 ± 13.34 b	95.14 ± 21.95 ab	97.04 ± 24.22 ab	111.03 ± 6.04 ab	123.03 ± 19.79 a
WEOC (mg kg^−1^)	151.90 ± 6.54 b	167.86 ± 18.05 ab	164.67 ± 11.16 ab	157.79 ± 7.67 b	180.74 ± 7.89 a
NO_3_^−^–N (mg kg^−1^)	1.09 ± 0.07 a	1.08 ± 0.05 a	1.23 ± 0.24 a	1.39 ± 0.55 a	1.33 ± 0.15 a
MBC (mg kg^−1^)	261.89 ± 75.36 a	320.22 ± 65.78 a	304.03 ± 87.39 a	314.10 ± 91.94 a	307.69 ± 59.11 a
MBN (mg kg^−1^)	17.71 ± 8.20 b	38.84 ± 6.59 a	39.30 ± 11.71 a	40.28 ± 9.97 a	43.19 ± 6.24 a
MBP (mg kg^−1^)	0.35 ± 0.11 b	2.41 ± 0.48 a	2.30 ± 0.80 a	0.66 ± 0.25 b	2.05 ± 0.01 a

Note: SOM, soil organic matter; TN, total nitrogen; TP, total phosphorus; AK, available potassium; AP, available phosphorus; AN, available nitrogen; WEOC, water-extractable organic carbon; MBC, microbial biomass carbon; MBN, microbial biomass nitrogen; MBP, microbial biomass phosphorus. Means ± standard errors (*n* = 4). Different letters indicate a significant difference among different treatments (Duncan, *p* < 0.05).

## Data Availability

The original contributions presented in this study are included in the article/Supplementary Material. Further inquiries can be directed to the corresponding authors.

## Supplementary Materials

The following supporting information can be downloaded at: https://www.mdpi.com/article/10.3390/microorganisms13112434/s1, Figure S1: Community structures of the top 10 most abundant taxa in bacterial (a), fungal (b), and archaeal (c) communities in soil; Figure S2: Correlations between microbial abundance, diversity, major microbial taxa (bacteria, fungi, and archaea), and soil properties; Figure S3: Classification of keystone taxa in the microbial co-occurrence network based on Zi and Pi connectivity metrics; Table S1: Application rates of different fertilizers (kg/ha); Table S2: Soil microbial community diversity index; Table S3: Composition of keystone taxa (network hubs and module hubs) in the co-occurrence network.
