# Supplementary material for: Organic and Inorganic Phosphorus Inputs Shape Wheat Productivity and Soil Bioavailability: A Microbial and Enzymatic Perspective from Long-Term Field Trials
Source: Microorganisms. 2025 Oct 23;13(11):2434. doi: 10.3390/microorganisms13112434 (PMC12654160; doi:10.3390/microorganisms13112434)
Supplement: Supplementary file 1 [file microorganisms-13-02434-s001.zip › microorganisms-3878143-supplementary.pdf]

Supplementary materials

Table S1. Application rates of different fertilizers (kg/ha)

Treatments	Total nutrient input			
	N	P_2O_5	K_2O	Straw
CK	——	——	——	——
OPT	165	90	120	——
OPTN	249	90	120	——
OPTP	165	135	120	——
OPTM	165	90	120	3000

Table S2. Soil microbial community diversity index

	Treatment	CK	OPT	OPTN	OPTP	OPTM
Bacteria	Sobs	4523.25±187.70 a	4516.25±76.72 a	4599.50±172.29 a	4613.00±174.10 a	4667.50±132.44 a
	Ace	5753.93±265.69 a	5825.32±112.51 a	5936.70±238.87 a	5937.48±197.35 a	5984.43±127.13 a
	Chao	5515.51±263.48 a	5590.07±123.13 a	5699.92±171.88 a	5704.38±140.62 a	5731.02±118.95 a
	Shannon	7.14±0.04 a	7.14±0.03 a	7.14±0.02 a	7.18±0.05 a	7.18±0.03 a
	Simpson	0.0024±0.0002 ab	0.0023±0.0001 ab	0.0025±0.0003 a	0.0022±0.0001 b	0.0023±0.0001 ab
Fungi	Sobs	724.25±90.47 a	765.00±181.90 a	805.50±140.88 a	916.75±205.07 a	909.25±166.78 a
	Ace	745.37±98.14 a	794.16±189.88 a	836.72±139.12 a	972.18±235.51 a	956.47±187.09 a
	Chao	748.55±99.97 a	799.20±190.66 a	846.07±149.36 a	979.66±229.25 a	960.44±187.08 a
	Shannon	4.54±0.26 a	4.75±0.20 a	4.81±0.32 a	4.88±0.24 a	4.72±0.30 a
	Simpson	0.0458±0.0207 a	0.0292±0.0040 a	0.0323±0.0131 a	0.0257±0.0115 a	0.0323±0.0209 a
Archaea	Sobs	295.75±20.02 a	288.50±35.30 a	292.75±21.16 a	284.25±14.50 a	285.75±11.59 a
	Ace	372.99±33.49 a	366.67±52.28 a	364.18±31.96 a	346.68±7.92 a	348.49±27.37 a
	Chao	363.06±34.52 a	362.39±62.87 a	363.13±29.27 a	341.35±2.05 a	340.87±29.96 a
	Shannon	2.69±0.09 a	2.55±0.16 ab	2.50±0.10 b	2.59±0.13 ab	2.58±0.07 ab
	Simpson	0.1948±0.0173 b	0.2291±0.0190 a	0.2469±0.0200 a	0.2204±0.0231 ab	0.2241±0.0116 ab

Table S3. Composition of keystone taxa (network hubs and module hubs) in the co-occurrence network

Network hubs	degree	Betweenness centrality	Zi	Pi	Taxonomy
OTU2795	34	896	2.68	0.63	Bacteria; g__Solirubrobacter
**Module hubs**	**degree**	**Betweenness centrality**	**Zi**	**Pi**	**Taxonomy**
OTU3409	45	12052	3.68	0.00	Bacteria; c__Thermodesulfovibrionia
OTU4641	36	10626	2.90	0.23	Bacteria; g__Marmoricola
OTU2382	8	3458	2.74	0.50	Bacteria; o__Vicinamibacterales
OTU2276	48	13207	4.01	0.00	Bacteria; g__MND1
OTU4021	38	5771	3.12	0.00	Bacteria; o__Gaiellales
OTU4851	14	4391	3.00	0.49	Bacteria; f__Anaerolineaceae
OTU2820	33	6135	2.57	0.00	Bacteria; g__MB-A2-108
OTU5291	33	1757	2.57	0.00	Bacteria; g__Gaiella
OTU3861	36	2639	2.90	0.36	Bacteria; c__MB-A2-108
OTU2709	16	4631	2.90	0.00	Bacteria; g__Flavobacterium
OTU4479	33	4271	2.57	0.00	Bacteria; f__67-14
OTU4380	13	2361	2.69	0.00	Bacteria; g__mle1-7
OTU581	18	6141	3.39	0.00	Archaea; f__Nitrosotaleaceae
OTU527	17	5273	3.17	0.62	Archaea; g__Candidatus_Nitrocosmicus
OTU532	18	11242	3.44	0.00	Archaea; g__Methanocella
OTU383	8	2144	2.74	0.00	Fungi; o__Agaricales
OTU3443	18	5696	3.44	0.00	Fungi; f__Hyaloscyphaceae
OTU194	16	3731	2.90	0.00	Fungi; k__Fungi
OTU21	8	5150	2.74	0.00	Fungi; k__Fungi

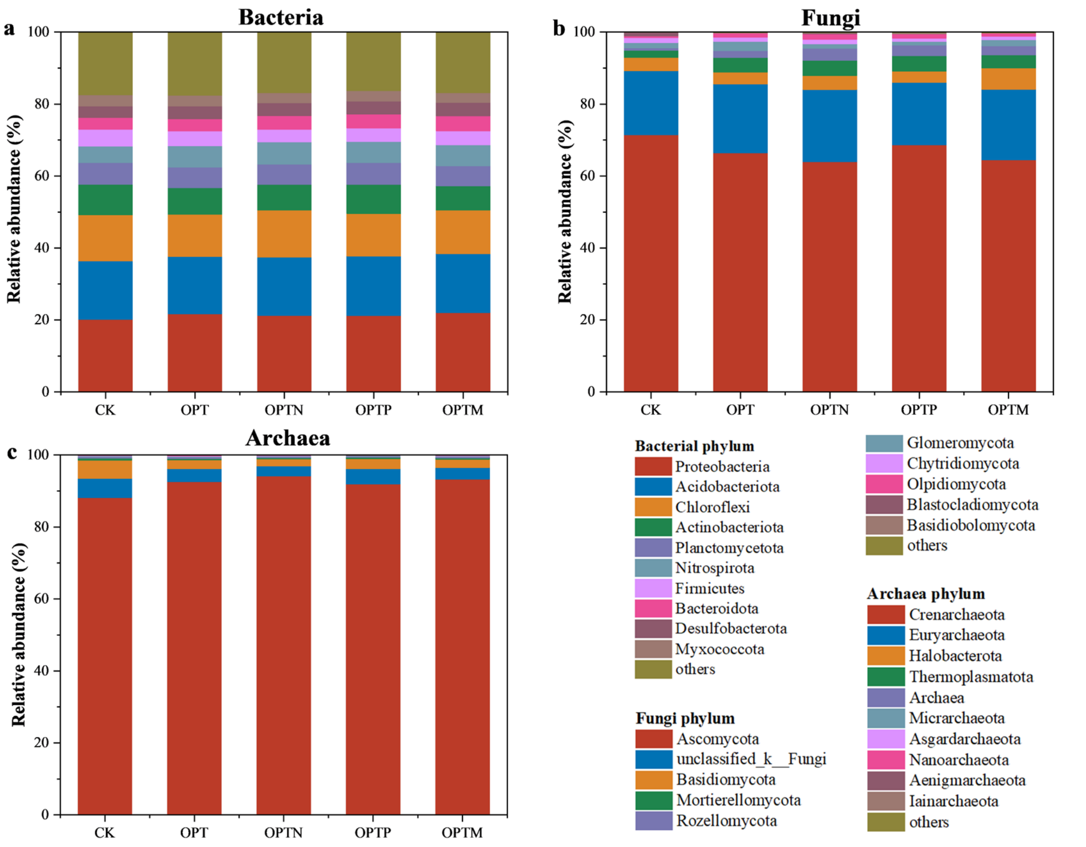

Figure S1. Community structures of the top 10 most abundant taxa in bacterial (a), fungal (b), and archaeal (c) communities in soil.

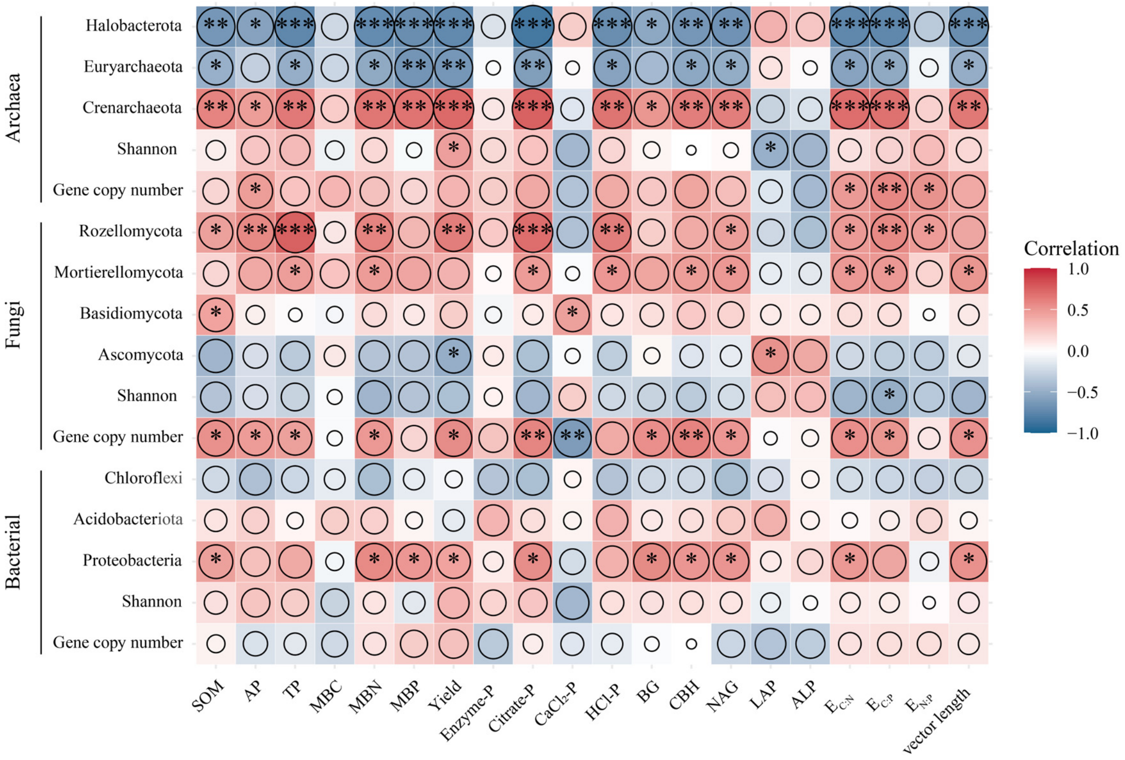

Figure S2. Correlations between microbial abundance, diversity, major microbial taxa (bacteria, fungi, and archaea), and soil properties. *, $P < 0.05$, **, $P < 0.01$, ***, $P < 0.001$.

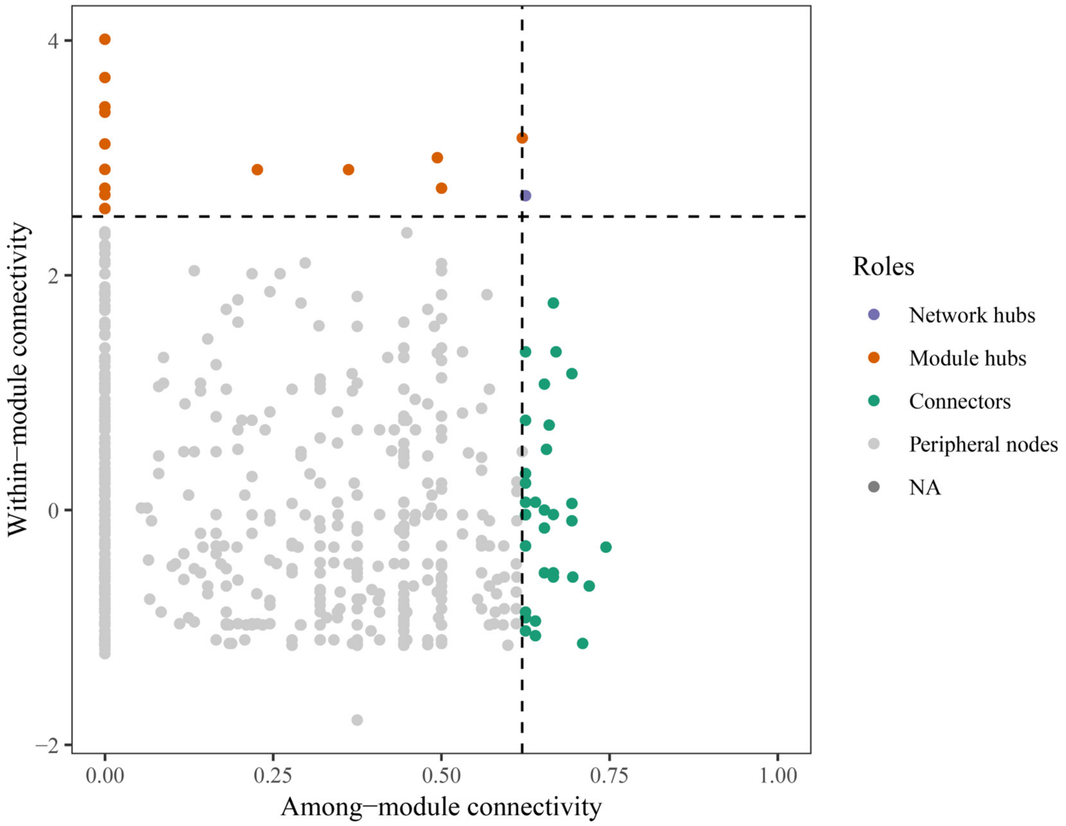

Figure S3. Classification of keystone taxa in the microbial co-occurrence network based on Zi and Pi connectivity metrics.
